# Thrombolytic proteins profiling: High‐throughput activity, selectivity, and resistance assays

**DOI:** 10.1002/2211-5463.70132

**Published:** 2025-10-04

**Authors:** Martin Toul, Alan Strunga, Jiri Damborsky, Zbynek Prokop

**Affiliations:** ^1^ Loschmidt Laboratories, Department of Experimental Biology and RECETOX, Faculty of Science Masaryk University Brno Czech Republic; ^2^ International Clinical Research Center St. Anne's University Hospital Brno Czech Republic; ^3^ Present address: Unit for Structural Biology, VIB Center for Inflammation Research (IRC) and Department of Biochemistry and Microbiology Ghent University Belgium

**Keywords:** alteplase, fibrinolysis, PAI‐1 inhibition resistance, plasminogen activator, staphylokinase, tenecteplase

## Abstract

Cardiovascular diseases, including thrombotic events such as ischemic stroke, pulmonary embolism, and myocardial infarction, are among the leading causes of morbidity and disability worldwide. The application of clot‐dissolving thrombolytic enzymes is a cost‐effective therapeutic intervention for these life‐threatening conditions. However, the effectiveness and safety profiles of current drugs are suboptimal, necessitating the discovery of new medicines or the engineering and enhancement of the existing ones. Here, we present a set of optimized biochemical protocols that allow robust screening and the therapeutic potential assessment of thrombolytic biomolecules. The assays provide information on multiple characteristics such as enzymatic activity, fibrinolysis rate, fibrin and fibrinogen stimulation, fibrin selectivity, clot binding affinity, and inhibition resistance. Such detailed characterization enables a rapid and reliable evaluation of candidate effectiveness and provides an indication of biological half‐life, associated bleeding complications, and other side effects. We demonstrate the credibility of the methodology by applying it to the two most widely used thrombolytic drugs: alteplase (Activase®/Actilyse®) and tenecteplase (Metalyse®/TNKase®). Consistent with previous studies, tenecteplase exhibited increased fibrin selectivity and inhibition resistance, which explains its extended half‐life. Our findings reinforce the growing consensus that tenecteplase may be a superior alternative to alteplase for thrombolytic treatment.

## Graphical abstract

We present optimized biochemical protocols for evaluating thrombolytic proteins, enabling rapid and robust screening of enzymatic activity, inhibition resistance, and fibrin affinity, stimulation, and selectivity. The outcome translates to key clinical indicators such as biological half‐life and bleeding risk. These assays streamline the development of safer and more effective clot‐dissolving therapies by guiding the design and engineering of next‐generation thrombolytics.

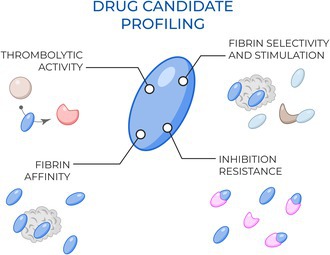

**Abbreviations**
AMC: 7‐amino‐4‐methylcoumarin
D‐VLK‐AMC: D‐Val‐Leu‐Lys‐(7‐amino‐4‐methylcoumarin)
D‐VLK‐pNA: D‐Val‐Leu‐Lys‐p‐nitroanilide
PAI‐1: plasminogen activator inhibitor‐1

Ischemic stroke, pulmonary embolism, and myocardial infarction are responsible for millions of deaths and disabilities every year, making them one of the deadliest diseases [1, 2]. They are caused by insufficient or no blood flow in the corresponding tissue due to pathological fibrin clots/thrombi occluding blood vessels (Fig. 1A) [3, 4]. Therapy based on drug‐induced thrombolysis represents a very convenient, easily accessible, and cost‐effective option as opposed to mechanical thrombectomy requiring specialized and highly trained medical personnel, hence limited to only certain large hospitals [5, 6]. Unfortunately, current thrombolytic drugs are not optimal and possess multiple side effects, making their further development critical to mitigate the increasing socioeconomic burden [7].

**Fig. 1:**
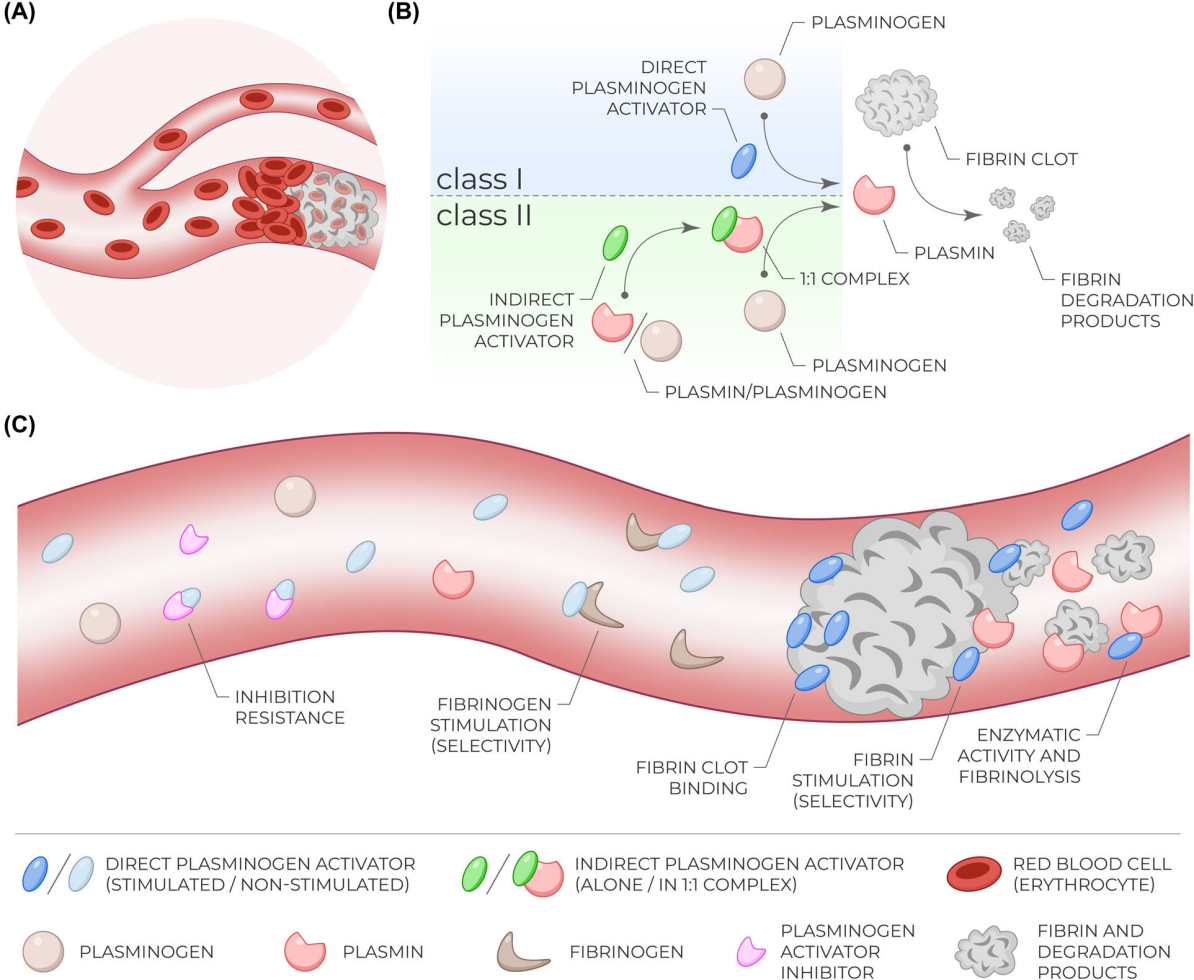
Molecular principles and regulations of thrombosis and thrombolysis. (A) Schematic illustration of a blood vessel occluded with a clot/thrombus, blocking blood flow and causing pathologies including ischemic stroke, pulmonary embolism, and myocardial infarction. (B) The molecular mechanism of the thrombolytic therapy is based on either direct plasminogen‐to‐plasmin conversion by a thrombolytic drug (direct plasminogen activator; *class I*) or on the preceding interaction of an indirect plasminogen activator with plasmin(ogen) to form a 1:1 complex capable of the same plasminogen‐to‐plasmin conversion (*class II*). Both events then lead to the common follow‐up of fibrin degradation by the action of generated plasmin. (C) Examples of important instances occurring in the bloodstream and significantly influencing the overall effectiveness and therapeutic potential of thrombolytics.

Two classes of thrombolytic proteins have been identified up to date—(i) direct plasminogen activators and (ii) indirect plasminogen activators (Fig. 1B) [8, 9]. Direct plasminogen activators (e.g., alteplase, tenecteplase, urokinase, and desmoteplase) possess enzymatic activities, so they can directly convert the zymogen called plasminogen into its active form plasmin that, in turn, cleaves the fibrin polymer, the main clumping component of a thrombus (Fig. 1B—*Class I*) [10]. In contrast, indirect plasminogen activators (e.g., streptokinase and staphylokinase) have no enzymatic activities at all, but their interactions with plasmin(ogen) modify the molecule's substrate specificity to activate other plasminogen molecules. Therefore, indirect plasminogen activators first form a 1:1 complex with either plasmin or plasminogen, resulting in a typical thrombolytic assembly capable of standard plasminogen‐to‐plasmin conversion (Fig. 1B—*Class II*) [11, 12]. The different modes of action of either thrombolytic class require different characteristics to be improved for the clinical application, necessitating different approaches and assays for their characterization [7, 13, 14, 15]. Although enzymatic and fibrinolysis activities can be determined for both thrombolytic classes in very similar ways with only minor modifications (e.g., concentrations and time windows), this protocol article primarily focuses on assays applicable to direct plasminogen activator thrombolytics (*Class I*).

Owing to the complex interplay of numerous factors in human bodies, the overall clinical potential, treatment efficacy, and safety of a particular thrombolytic candidate is given not only by its thrombolysis rate but also by multiple other properties (Fig. 1C) [9, 16, 17, 18]. The ideal thrombolytic is desired to be nearly inactive when freely circulating in the bloodstream or in contact with nonpolymerized fibrinogen, while the presence of a thrombus containing polymerized fibrin should massively stimulate its activity. Such behavior ensures selective hydrolysis of pathological clots while preventing unspecific plasminogen activation across the whole body, which is associated with bleeding complications [19, 20]. Similarly, the ideal thrombolytic is resistant to plasminogen activator inhibitors (PAIs), namely PAI‐1, to prolong its half‐life [21, 22]. Finally, the desired massive activity stimulation by fibrin should not be associated with too tight fibrin clot binding in order to ensure its effective penetration and clot lysis throughout the whole volume [17, 23, 24]. All these characteristics—that is, enzymatic activity (plasminogen activation and fibrinolysis rate), fibrin and fibrinogen stimulation, fibrin selectivity, clot binding, and inhibition resistance (Fig. 1C)—need to be considered when globally evaluating the clinical relevance of tested molecules [7, 15, 16]. In this protocol, we present a comprehensive summary of optimized assays allowing for robustly screening and assessing the therapeutic potential of candidate thrombolytic proteins by quantifying all the aforementioned thrombolytic characteristics. The outcome of the assays serves as a decisive selection platform for any follow‐up *in vivo* trials in animal models.

## Method principle

### Enzymatic activity assay

The value of enzymatic activity determines how quickly a tested thrombolytic converts plasminogen to plasmin, that is, to a molecule responsible for fibrin clot lysis. Therefore, it is a key indicator of clot lysis effectiveness. The activity assay utilizes the fluorogenic substrate D‐VLK‐AMC (or alternatively the chromogenic substrate D‐VLK‐pNA), which is hydrolyzed by plasmin. The specific recognition is secured by the tripeptide Val‐Leu‐Lys (VLK), which mimics the cleavage site in fibrin while the aminomethylcoumarin (AMC) conjugate allows convenient monitoring of the enzymatic activity via fluorescence measurement. Upon the enzymatic cleavage, a free AMC fluorophore is released, resulting in increased fluorescence [7, 13].

**Fig. 2:**
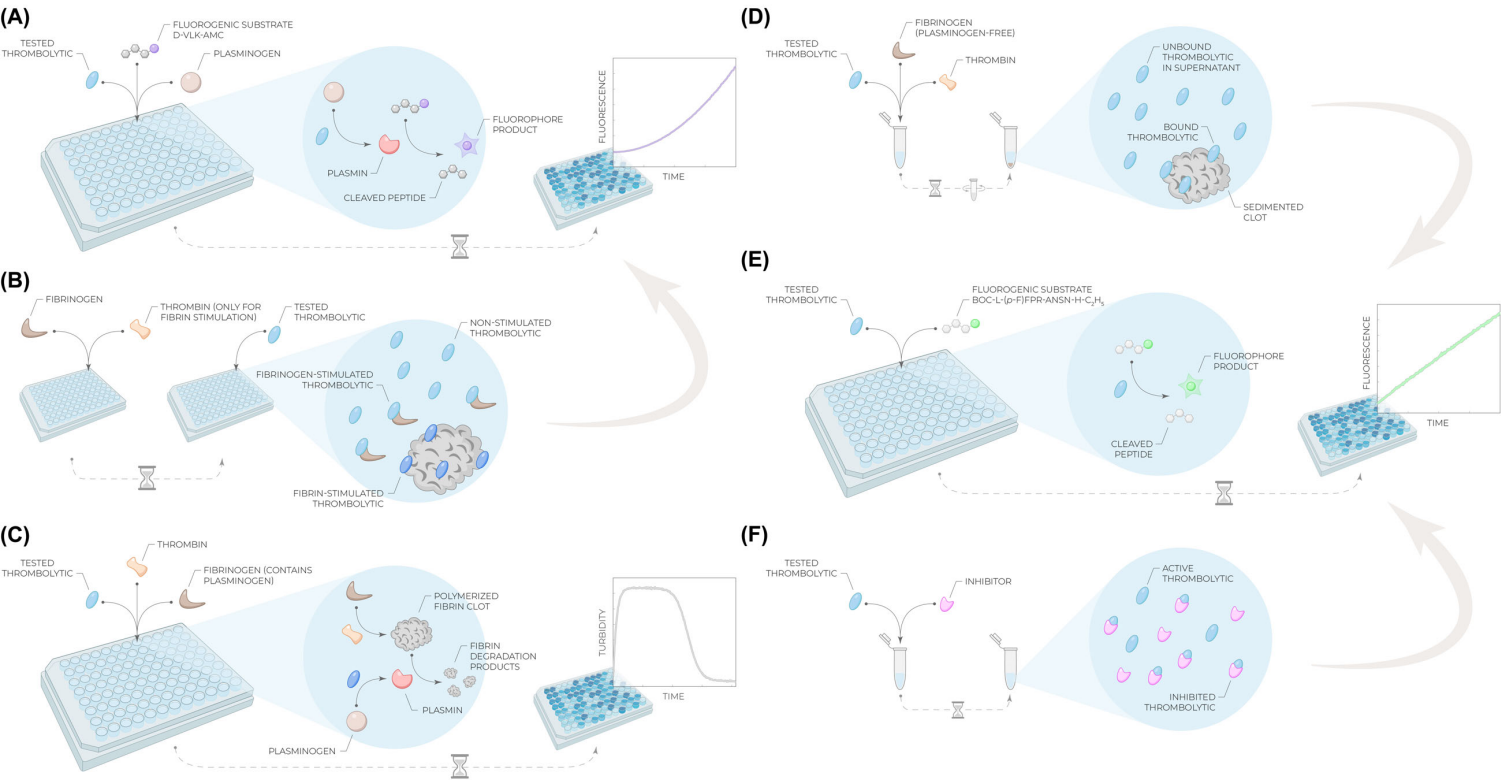
Summary of assays described in this protocol and their principles. (A) *Enzymatic activity assay* is based on mixing a tested thrombolytic with plasminogen and plasmin fluorogenic substrate D‐VLK‐AMC, resulting in a cascade of two reactions: plasminogen‐to‐plasmin conversion by the tested thrombolytic, followed by D‐VLK‐AMC hydrolysis by generated plasmin, yielding fluorescence increase. Due to the coupled nature, the signal gives a parabolic increase over time (Eq. 3). (B) *Fibrin(ogen) stimulation and selectivity assay* compares enzymatic activities in three different conditions: absence of stimulant, presence of fibrinogen stimulant, and presence of fibrin stimulant prepared by mixing thrombin and fibrinogen. The actual activities are then measured via the setup shown in (A), and stimulation/selectivity factors are calculated according to their definitions [25, 26]. (C) *Fibrinolysis activity assay* utilizes plasma‐isolated fibrinogen formulation, which typically contains traces of plasminogen. Upon mixing it with the tested thrombolytic and thrombin, clotting and fibrin formation can be observed, followed by the fibrinolysis phase due to the action of the tested thrombolytic. The time required to reach 50% lysis (*t*_50_) is the decisive factor determining the efficiency of fibrinolysis. (D) *Fibrin binding assay* requires plasminogen‐free fibrinogen to prevent any clot lysis. When mixed with thrombin and the tested thrombolytic, a fibrin clot is formed with the thrombolytic being in an equilibrium between the free/unbound state in solution and the bound state in/on the fibrin clot. After the centrifugation and clot separation, the amount of unbound thrombolytic in the supernatant is measured via the assay described in (E) to determine the binding extent. (E) Direct thrombolytic fluorogenic substrate Boc‐L‐(*p*‐F)FPR‐ANSN‐H‐C_2_H_5_ is directly hydrolyzed by thrombolytic enzymes such as alteplase, urokinase, tenecteplase, or desmoteplase. The reaction releases free fluorophore, resulting in a linear increase of the fluorescence signal over time with the slope proportional to the activity and amount of the tested thrombolytic. (F) *Inhibition resistance assay* is based on preincubation of the tested thrombolytic with the inhibitor of interest, resulting in an equilibrium between the free active thrombolytic and the inhibitor‐bound inactive fraction. The residual activity is then measured via the assay illustrated in (E) to determine the extent of inhibition.

By mixing the fluorogenic substrate D‐VLK‐AMC with plasminogen and a tested thrombolytic, a cascade of two reactions is initiated: (i) the tested thrombolytic enzyme converts plasminogen to plasmin and subsequently (ii) generated plasmin converts the fluorogenic substrate D‐VLK‐AMC to the fluorescent product AMC (Fig. 2A). This means that increased enzymatic activity of the tested thrombolytic leads to the faster plasmin production, hence also faster AMC production, ultimately observed as a faster fluorescence signal increase. Due to the coupled nature of the assay, the signal increases with time in a parabolic manner (Eq. 3) as described and derived previously [13]. As a result, this assay provides a very robust and reliable determination of the tested thrombolytic enzyme activity in terms of the plasminogen‐to‐plasmin conversion.

### Fibrin(ogen) stimulation and selectivity assay

Many thrombolytic enzymes can be stimulated by various plasma factors, namely fibrinogen and fibrin. For this purpose, three principal parameters have been defined to quantitatively describe the stimulation extent of various thrombolytics: (i) fibrinogen stimulation factor, (ii) fibrin stimulation factor, and (iii) fibrin selectivity [25, 26]. Fibrinogen and fibrin stimulation factors are defined as ratios of enzymatic activities in the presence of the corresponding stimulant over activities in the absence of the stimulant. Therefore, the factor defines the relative fold activity increase upon stimulation. The fibrin selectivity factor is then defined as the ratio of fibrin‐stimulated activity over fibrinogen‐stimulated activity. A high fibrin stimulation factor and low fibrinogen stimulation factor (hence high fibrin selectivity) is the most desired scenario because it makes the activation of plasminogen more targeted by taking place only in the proximity of pathological fibrin clots. This results in significantly attenuated side effects associated with fibrinogenolysis and systemic plasminemia [27], justifying the importance of determining these parameters [28].

Experimentally, stimulated enzymatic activities are measured and determined in the same way as described above in Section Enzymatic activity assay, but in individual microplate wells where either fibrinogen or fibrin is present (Fig. 2B). The presence of fibrinogen is achieved very simply by directly adding it into the reaction mixture containing the tested thrombolytic, plasminogen, and D‐VLK‐AMC. However, the fibrin stimulation experiment requires prior polymerization and clot formation. This is accomplished by mixing small volumes of fibrinogen and thrombin inside a microplate well, followed by a short incubation time [26]. Once the polymerized fibrin clot is formed, the same well is supplied with the reaction mixture containing the tested thrombolytic, plasminogen, and D‐VLK‐AMC.

### Fibrinolysis activity assay

Although enzymatic activity and selectivity values are very useful for understanding and assessment of the thrombolytic molecular principles and their follow‐up engineering, real clot fibrinolysis is a complex interplay of multiple factors. Therefore, it is highly desired to combine the aforementioned assays with the fibrinolysis activity experiment [29]. In this setup, a global assessment of thrombolytic efficiency can be made using real fibrin‐containing, yet still reproducibly prepared clots.

The assay involves a simple mixing of the tested thrombolytic with thrombin, followed by the addition of fibrinogen to initiate the reaction (Fig. 2C). The coagulation and fibrinolysis progress are then monitored via measuring turbidity at 405 nm, which is proportional to the amount of the macroscopic polymerized fibrin clot: The signal initially increases as the clot is being formed but subsequently starts decreasing due to the fibrinolysis induced by the tested thrombolytic protein. The time required to reach 50% clot lysis (*t*_50_) is the decisive factor determining the fibrinolysis efficiency of the tested thrombolytic [30]. It can be quantitatively determined by fitting the fibrinolysis phase of the collected curve using a sigmoid equation (Eq. 4).

### Fibrin binding assay

Another limiting factor of current thrombolytics is their insufficient clot penetrability. The proteins typically exhibit high fibrin affinity, which is essential for efficient recognition of the targeted clot; but as a drawback, thrombolytic proteins then stick to the clot surface, lysing it very slowly in surface layers [24, 31]. Ideally, fibrin affinity should be decreased while retaining unimpaired fibrin selectivity described in the previous Section Fibrin(ogen) stimulation and selectivity assay, making it critical to determine both these parameters for rational and well‐informed thrombolytic assessment. The ideal scenario of low fibrin affinity and high fibrin selectivity would improve clot penetrability while preserving enzymatic activity, making the overall thrombolysis process much more effective by lysing clots from inside at all hydrolytic cleavage sites simultaneously [9].

In the fibrin clot binding assay, plasminogen‐free fibrinogen at various concentrations is premixed with a tested thrombolytic molecule, followed by the addition of thrombin to initiate fibrinogen polymerization and the generation of macroscopic fibrin clots. In such an arrangement, no fibrin cleavage occurs thanks to the absence of plasminogen, allowing the dissection of the fibrin binding parameter from fibrinolysis. After the incubation for a defined time, the clot is centrifuged to separate the bound fraction of thrombolytics while the supernatant is collected and tested for the amount of unbound thrombolytic (Fig. 2D) [32]. This is achieved by supplementing the supernatant with the direct fluorogenic substrate Boc‐L‐(*p*‐F)FPR‐ANSN‐H‐C_2_H_5_ (or alternatively the chromogenic substrate H‐D‐IPR‐pNA). This substrate is analogical to D‐VLK‐AMC described in Section Enzymatic activity assay but contains another tripeptide sequence (Phe/Ile‐Pro‐Arg), which is specifically recognized directly by thrombolytic enzymes such as alteplase, urokinase, tenecteplase, or desmoteplase (Fig. 2E). Cleavage of this substrate provides a fluorescence signal, which increases linearly over time, with the slope proportional to the amount of the unbound thrombolytic enzyme in the supernatant [7]. The dependence of the unbound thrombolytic amount on the fibrin concentration provides a typical hyperbolic trend which can be fit using Eq. 6 to determine the dissociation constant *K*_d_ (inversed value of the affinity) for the fibrin–thrombolytic interaction.

### Inhibition resistance assay

Inactivation of thrombolytic enzymes by inhibitors circulating in the human bloodstream, namely plasminogen activator inhibitor‐1 (PAI‐1), is one of the main factors quickly impairing the therapeutic action of administered thrombolytics and significantly decreasing their biological half‐life [33]. Therefore, determining the thrombolytic inhibition resistance parameter and selecting the most resistant one is critical for the success of further preclinical testing. This characteristic is quantitatively described by the parameter IC_50_, which corresponds to the concentration of inhibitor providing the reduction in enzymatic activity to 50% [34].

Experimental measurement of the PAI‐1 resistance is relatively straightforward and analogous to any other typical enzymology inhibition experiment. A tested thrombolytic enzyme is preincubated with various concentrations of an inhibitor (Fig. 2F), and the residual activity is determined by adding the same direct fluorogenic substrate Boc‐L‐(*p*‐F)FPR‐ANSN‐H‐C_2_H_5_ as described in the previous Section Fibrin binding assay (Fig. 2E) [35]. The slope of the fluorescence increase over time is proportional to the amount of the active thrombolytic enzyme, that is, to the residual activity after the incubation with PAI‐1. The dependence of residual activity on the inhibitor concentration provides a classical inhibition pattern, which can be fit using the previously derived equation for a tightly bound inhibitor (Eq. 7) to determine the IC_50_ value [36].

## Materials

### Enzymatic activity assay

1. Tested thrombolytic enzyme
2. Human plasminogen (e.g., #10874477001 from Roche [Mannheim, Germany]; > 5 μm stock)
3. Plasmin fluorogenic substrate D‐VLK‐AMC (e.g., #13201 from AAT Bioquest [Sunnyvale, CA, USA]; typically 20 mm stock in DMSO)
4. Assay buffer: PBS pH 7.4 (10 mm Na_2_HPO_4_, 1.8 mm KH_2_PO_4_, 2.7 mm KCl, 137 mm NaCl) supplemented with 1 mm CaCl_2_ and 0.01% Tween 80
5. Black 96‐well clear‐bottom microplate (e.g., #6005029 from PerkinElmer [Waltham, MA, USA])
6. Self‐adhesive sealing film for microplates (e.g., #Z688630 from Merck [Darmstadt, Germany])
7. Microplate reader (spectrophotometer) capable of fluorescence readout, temperature‐controlled incubation, and shaking (see *Tips and Tricks* if some parameters are unavailable)
8. *Optional*: 7‐amino‐4‐methylcoumarin (AMC) standard (e.g., #257370 from Sigma‐Aldrich [Saint Louis, MO, USA]; typically 50 mm stock in DMSO)

### Fibrin(ogen) stimulation and selectivity assay

1. Tested thrombolytic enzyme
2. Human fibrinogen (e.g., #F3879 from Sigma‐Aldrich [Saint Louis, MO, USA]; > 15 mg·mL^−1^ stock)
3. Human thrombin (e.g., #T6884 from Sigma‐Aldrich [Saint Louis, MO, USA]; > 2 UN·mL^−1^ stock)
4. Human plasminogen (e.g., #10874477001 from Roche [Mannheim, Germany]; > 5 μm stock)
5. Plasmin fluorogenic substrate D‐VLK‐AMC (e.g., #13201 from AAT Bioquest [Sunnyvale, CA, USA]; typically 20 mm stock in DMSO)
6. Assay buffer: PBS pH 7.4 (10 mm Na_2_HPO_4_, 1.8 mm KH_2_PO_4_, 2.7 mm KCl, 137 mm NaCl) supplemented with 1 mm CaCl_2_ and 0.01% Tween 80
7. Black 96‐well clear‐bottom microplate (e.g., #6005029 from PerkinElmer [Waltham, MA, USA])
8. Self‐adhesive sealing film for microplates (e.g., #Z688630 from Merck [Darmstadt, Germany])
9. Microplate reader (spectrophotometer) capable of fluorescence readout, temperature‐controlled incubation, and shaking (see *Tips and Tricks* if some parameters are unavailable)

### Fibrinolysis activity assay

1. Tested thrombolytic enzyme
2. Human fibrinogen (e.g., #F3879 from Sigma‐Aldrich [Saint Louis, MO, USA]; > 10 mg·mL^−1^ stock)
3. Human thrombin (e.g., #T6884 from Sigma‐Aldrich [Saint Louis, MO, USA]; > 1 UN·mL^−1^ stock)
4. Assay buffer: PBS pH 7.4 (10 mm Na_2_HPO_4_, 1.8 mm KH_2_PO_4_, 2.7 mm KCl, 137 mm NaCl) supplemented with 1 mm CaCl_2_ and 0.01% Tween 80
5. Any standard 96‐well microplate
6. Microplate reader (spectrophotometer) capable of absorbance readout, temperature‐controlled incubation, and shaking (see *Tips and Tricks* if some parameters are unavailable)

### Fibrin binding assay

1. Tested thrombolytic enzyme
2. Human ‘plasminogen‐free’ fibrinogen (e.g., #PP002C from HYPHEN Biomed. [Neuville‐sur‐Oise, France]; > 50 μm stock)
3. Human thrombin (e.g., #T6884 from Sigma‐Aldrich [Saint Louis, MO, USA]; > 20 UN·mL^−1^ stock)
4. Direct thrombolytic fluorogenic substrate Boc‐L‐(*p*‐F)FPR‐ANSN‐H‐C_2_H_5_ (e.g., #T5600‐19 from US Biological [Salem, MA, USA]; typically 10 mm stock in DMSO)
5. Assay buffer: PBS pH 7.4 (10 mm Na_2_HPO_4_, 1.8 mm KH_2_PO_4_, 2.7 mm KCl, 137 mm NaCl) supplemented with 1 mm CaCl_2_ and 0.01% Tween 80
6. Cooled Eppendorf tube centrifuge
7. Black 96‐well clear‐bottom microplate (e.g., #6005029 from PerkinElmer [Waltham, MA, USA])
8. Self‐adhesive sealing film for microplates (e.g., #Z688630 from Merck [Darmstadt, Germany])
9. Temperature‐controlled shaking incubator
10. Microplate reader (spectrophotometer) capable of fluorescence readout, temperature‐controlled incubation, and shaking (see *Tips and Tricks* if some parameters are unavailable)

### Inhibition resistance assay

1. Tested thrombolytic enzyme
2. Human plasminogen activator inhibitor‐1 (PAI‐1; e.g., #A811 from Sigma‐Aldrich [Saint Louis, MO, USA]; > 2.5 μm stock)
3. Direct thrombolytic fluorogenic substrate Boc‐L‐(*p*‐F)FPR‐ANSN‐H‐C_2_H_5_ (e.g., #T5600‐19 from US Biological [Salem, MA, USA]; typically 10 mm stock in DMSO)
4. Assay buffer: PBS pH 7.4 (10 mm Na_2_HPO_4_, 1.8 mm KH_2_PO_4_, 2.7 mm KCl, 137 mm NaCl) supplemented with 1 mm CaCl_2_ and 0.01% Tween 80
5. Black 96‐well clear‐bottom microplate (e.g., #6005029 from PerkinElmer [Waltham, MA, USA])
6. Self‐adhesive sealing film for microplates (e.g., #Z688630 from Merck [Darmstadt, Germany])
7. Temperature‐controlled shaking incubator
8. Microplate reader (spectrophotometer) capable of fluorescence readout, temperature‐controlled incubation, and shaking (see *Tips and Tricks* if some parameters are unavailable)

## Methods

### Enzymatic activity assay

**Fig. 3:**
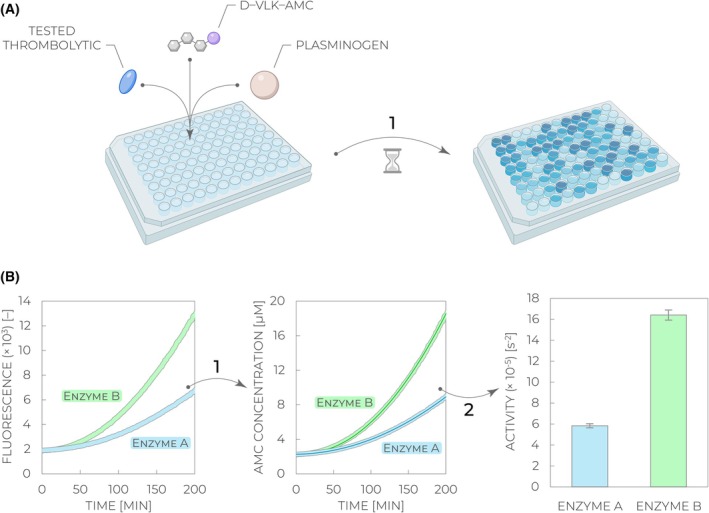
Workflow of the enzymatic activity assay. (A) A tested thrombolytic is mixed with its substrate plasminogen and the coupled fluorogenic substrate D‐VLK‐AMC, followed by incubation and monitoring of the fluorescence signal increase with time (*Step 1*). (B) Collected fluorescence kinetic curves are optionally recalculated to concentration changes of the 7‐amino‐4‐methylcoumarin (AMC) product formation based on the calibration curve measured with the AMC standard (*Step 1*). The kinetic data are fit (solid line) with the quadratic equation (Eq. 3) to obtain values of quadratic slopes, corresponding to enzymatic activities (*Step 2*). Relative differences in the derived values can be used to compare enzymatic activities of various thrombolytic enzymes (enzyme A vs. enzyme B).

1. Pre‐incubate the microplate reader to 37 °C and preset all the parameters to have it ready for data collection once the reaction is initiated—excitation 340 nm, emission 440 nm, bottom‐read position, measurement interval 60 s, total measurement time 2–4 h.
2. Dilute the starting material using the assay buffer to obtain 5 μm plasminogen, 800 μm D‐VLK‐AMC, and ~3 nm thrombolytic enzyme.
3. Prepare “Master Mix” by mixing plasminogen and D‐VLK‐AMC in a 2:1 ratio (e.g., 4900 μL of plasminogen and 2450 μL of D‐VLK‐AMC per one full plate).
4. Add 25 μL of the tested thrombolytic enzyme into a microplate well. Add each tested thrombolytic to at least three wells to have technical replicates.
5. Initiate the reaction by adding 75 μL of the Master Mix and immediately proceed with the next steps to start data collection as quickly as possible after the addition. The resulting concentrations in the well will be 2.5 μm plasminogen, 200 μm D‐VLK‐AMC, and ~0.75 nm thrombolytic.
6. Seal the microplate with an adhesive film to prevent evaporation.
7. Insert the sealed microplate into the microplate reader and start data collection with the parameters set in *Step 1* (Fig. 3A).
8. Analyze collected data and determine enzymatic activities using the corresponding method described in Section Kinetic analysis of plasminogen activation data. (Fig. 3B). Semi‐automated data analysis can be performed using File S1. Alternatively, “ZymogenActnCL” online tool within the Shiny App framework can be applied to perform the same type of analysis [30].

### Fibrin(ogen) stimulation and selectivity assay

**Fig. 4:**
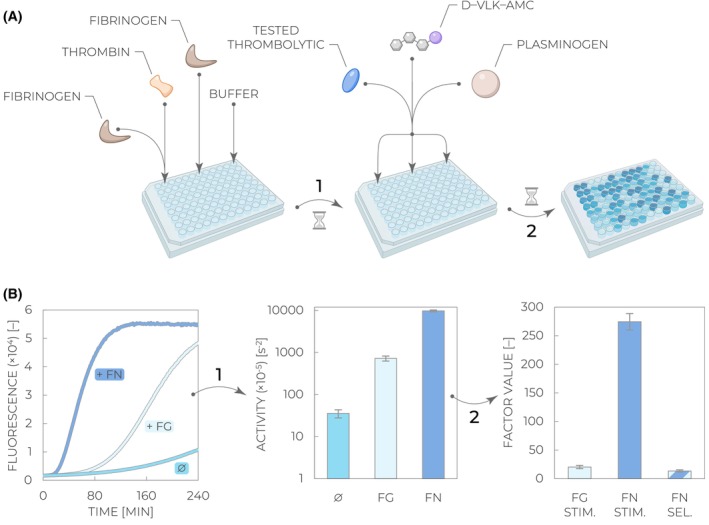
Workflow of the fibrin(ogen) stimulation and selectivity assay. (A) Fibrin‐stimulated activity is measured by premixing low volumes of fibrinogen and thrombin on the wall of a microplate well to generate small stimulating clots. Fibrinogen‐stimulated activity involves the prior addition of fibrinogen, and the absence of any stimulant is achieved by adding the reaction buffer only. Once fibrin clots are generated upon incubation (*Step 1*), all microplate wells are supplied with a tested thrombolytic, its substrate plasminogen, and the coupled fluorogenic substrate D–VLK–AMC. The reaction is monitored via the fluorescence increase with time (*Step 2*). (B) Collected kinetic curves in the absence of any stimulant (Ø) and in the presence of fibrinogen (Fg) and fibrin (Fn) are analyzed as described in Section Kinetic analysis of plasminogen activation data and schematically illustrated in Fig. 3B to determine enzymatic activity values (*Step 1*). These values are subsequently used for the calculation of fibrinogen stimulation (Fg stim.), fibrin stimulation (Fn stim.), and fibrin selectivity (Fn sel.) factors (*Step 2*) according to their definitions [25, 26].

1 Pre‐incubate the microplate reader to 37 °C and preset all the parameters to have it ready for data collection once the reaction is initiated—excitation 340 nm, emission 440 nm, bottom‐read position, measurement interval 60 s, total measurement time 2–4 h.
2 Dilute the starting material using the assay buffer to obtain 13 mg·mL^−1^ fibrinogen (~38 μm), 2 UN·mL^−1^ thrombin, 5 μm plasminogen, 800 μm D‐VLK‐AMC, and ~5 nm thrombolytic enzyme.
3 Prepare three different reaction conditions in separate microplate wells in at least three technical replicates (at least nine microplate wells in total):
  - No stimulation: Add 10 μL of the assay buffer into the microplate well.
  - Fibrinogen stimulation: Mix 8 μL of fibrinogen and 2 μL of the assay buffer inside a microplate well.
  - Fibrin stimulation: Mix 8 μL of fibrinogen and 2 μL of thrombin at the well “corner” (where the base meets the vertical wall) to form a fibrin clot. This is to avoid later interference of fluorescence measurements in the center of the well.
1 Seal the microplate with an adhesive film to prevent evaporation and incubate at room temperature for 60 min to let fibrinogen polymerize.
2 In the meantime, prepare ‘Master Mix’ by mixing plasminogen and D‐VLK‐AMC in a 2:1 ratio (e.g., 4900 μL of plasminogen and 2450 μL of D‐VLK‐AMC per one full plate).
3 After the incubation time, remove the sealing film, add 15 μL of the tested thrombolytic enzyme into each microplate well, and initiate the reaction by adding 75 μL of the Master Mix. Make sure you do not touch the preformed fibrin clots and immediately proceed with the next steps to start data collection as quickly as possible after the addition of all the components. The resulting concentrations in the wells will be 2.5 μm plasminogen, 200 μm D‐VLK‐AMC, ~0.75 nm thrombolytic, ~3 μm fibrin (where applicable), ~3 μm fibrinogen (where applicable), and 40 mUN·mL^−1^ thrombin (where applicable).
4 Seal the microplate with an adhesive film to prevent evaporation.
5 Insert the sealed microplate into the microplate reader and start data collection with the parameters set in *Step 1* (Fig. 4A).
6 Determine enzymatic activities at all three experimental conditions using the corresponding method described in Section Kinetic analysis of plasminogen activation data. Semi‐automated data analysis can be performed using File S1.
7 Calculate the final stimulation and selectivity parameters using the following formulae based on their definitions (Fig. 4B) [25, 26]:
  - Fibrinogen stimulation factor = ratio of “fibrinogen stimulation activity” over “no stimulation activity”
  - Fibrin stimulation factor = ratio of “fibrin stimulation activity” over “no stimulation activity”
  - Fibrin selectivity factor = ratio of “fibrin stimulation activity” over “fibrinogen stimulation activity”

### Kinetic analysis of plasminogen activation data

1. *Optional*: Generate the AMC product calibration curve. The concentration dependence of the AMC fluorescence *F* typically exhibits an exponential trend, so fit the points using Eq. 1 and use the obtained calibration parameters *F*_a_, *F*_b_, and *F*_c_ to recalculate fluorescence kinetic curves to AMC product concentration changes using Eq. 2. This step allows the determination of absolute activity values but is not required for relative activity comparison among tested thrombolytic variants.
2. Fit the initial “quadratic” phase of the fluorescence F or AMC concentration [AMC] increase (if recalculated in *Step 1*) using the second‐order polynomial Eq. 3 to determine the rate of the enzymatic reaction *v*_0_ (Fig. 3B) [13]. Offset *c* corresponds to the background spectroscopic signal, and *t*_0_ should roughly correspond to the dead time between the reaction initiation and the start of the measurement. Alternatively, you can plot kinetic data as fluorescence vs. time squared (*t* ^2^) and then do a simple linear fitting of the initial phase, where the slope corresponds to the desired enzymatic rate *v*_0_.
3. Discard any obvious outlier values and then average rates obtained for the replicates.
4. Use the final obtained values for variants comparison, stimulation/selectivity factors calculation, concentration dependence analysis, or any other purpose meeting your needs. Note that the whole analysis procedure can be conveniently performed in a semi‐automated manner using File S1. Alternatively, “ZymogenActnCL” online tool within the Shiny App framework can be applied to perform the same type of analysis [30].

(1) $$F=F_{a}\cdot \left(1-e^{-F_{b}\cdot \left[\mathrm{AMC}\right]}\right)+F_{c}$$

(2) $$\left[\mathrm{AMC}\right]=-\frac{1}{F_{b}}\cdot \ln\left(1-\frac{F-F_{c}}{F_{a}}\right)$$

(3) $$F\;\text{or}\;\left[\mathrm{AMC}\right]=v_{0}\cdot {\left(t+t_{0}\right)}^{2}+c$$

### Fibrinolysis activity assay

**Fig. 5:**
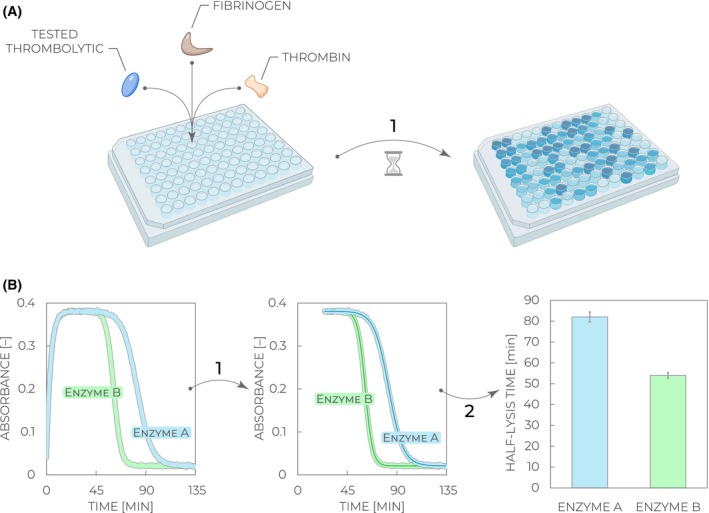
Workflow of the fibrinolysis activity assay. (A) A tested thrombolytic is mixed with thrombin and fibrinogen (containing a residual amount of plasminogen), followed by incubation and monitoring of the absorbance signal change with time (*Step 1*). (B) Collected absorbance kinetic curves are trimmed to exclude the initial clotting phase and keep only the sigmoid part of the curves (*Step 1*). The treated data with the plateau and the follow‐up fibrinolysis phase are fit (solid line) using the sigmoid equation (Eq. 4) to obtain values of the time required to reach 50% clot lysis, that is, half‐lysis time *t*_50_ (*Step 2*). Relative differences in the derived values can be used to compare fibrinolysis efficiencies of various thrombolytic enzymes (enzyme A vs. enzyme B).

1. Pre‐incubate the microplate reader to 37 °C and preset all the parameters to have it ready for data collection once the reaction is initiated—absorbance at 405 nm, measurement interval 30 s, total measurement time 2–4 h, shaking at 300 rpm.
2. Dilute the starting material using the assay buffer to obtain 10 mg·mL^−1^ fibrinogen (~29 μm), 1 UN·mL^−1^ thrombin, and ~10 nm thrombolytic enzyme.
3. Mix 50 μL of thrombin with 5 μL of the tested thrombolytic inside a microplate well. Prepare this mixture in at least three separate microplate wells to have technical replicates.
4. Initiate the reaction by adding 50 μL of fibrinogen and immediately proceed with the next step to start data collection as quickly as possible after the addition. The resulting concentrations in the well will be 0.5 UN·mL^−1^ thrombin, ~14 μm fibrinogen, and ~0.5 nm thrombolytic.
5. Insert the sealed microplate into the microplate reader and start data collection with the parameters set in *Step 1* (Fig. 5A).
6. Trim initial data points (absorbance *A*_405_ increase), corresponding to the clotting phase, and fit the following plateau and the fibrinolysis phase (absorbance *A*_405_ decrease) using the sigmoid Eq. 4, where *A*_p_ corresponds to the initial absorbance of the plateau phase, *A*_a_ to the amplitude of the absorbance decrease, *k* to the fibrinolysis rate, and *t*_50_ to the time of reaching 50% clot lysis. The value of *t*_50_ determines the fibrinolysis efficiency—the lower the value, the more effective fibrinolysis is achieved (Fig. 5B). Alternatively, the “ClotLysisCL” online tool within the Shiny App framework can be applied to obtain the value of *t*_50_ without the need for initial data trimming [30].

(4) $$A_{405}=A_{p}-\frac{A_{a}}{1+e^{-k\cdot \left(t-t_{50}\right)}}$$

### Fibrin binding assay

**Fig. 6:**
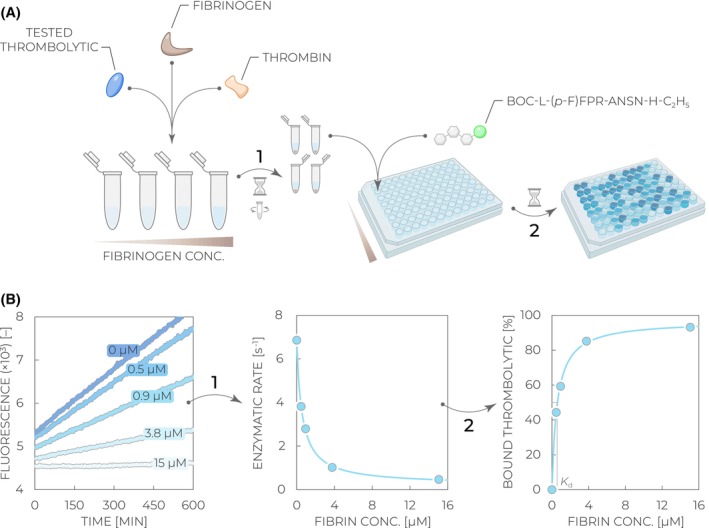
Workflow of the fibrin binding assay. (A) A tested thrombolytic is mixed with increasing fibrinogen concentrations, and the clotting is initiated by the addition of thrombin (*Step 1*). After the incubation, the samples are centrifuged, and the supernatants containing the unbound thrombolytic fraction are mixed with the direct fluorogenic substrate Boc‐L‐(*p*‐F)FPR‐ANSN‐H‐C_2_H_5_. The reaction is monitored via the fluorescence increase with time (*Step 2*). (B) Collected fluorescence kinetic curves at varied fibrin concentrations (dark to light blue) are used to determine residual activities (slopes of the fluorescence increase) and plotted against the used fibrin concentration (*Step 1*). The activity values are subsequently recalculated to relative amounts of bound thrombolytic (Eq. 5), and the desired value of the fibrin dissociation constant *K*_d_ is obtained by fitting the concentration dependence with the hyperbolic equation (Eq. 6).

1. Pre‐incubate the microplate reader to 37 °C and preset all the parameters to have it ready for data collection once the reaction is initiated—excitation 350 nm, emission 470 nm, bottom‐read position, measurement interval 60 s, total measurement time 2–4 h.
2. Dilute the starting material using the assay buffer to obtain 20 UN·mL^−1^ thrombin, 250 μm Boc‐L‐(*p*‐F)FPR‐ANSN‐H‐C_2_H_5_, and ~1.5 μm thrombolytic enzyme. Prepare a dilution series of fibrinogen ranging from 0 to 50 μm (at least six concentration points).
3. For each fibrinogen concentration, add 320 μL into separate Eppendorf tubes and mix each sample with 40 μL of the tested thrombolytic.
4. Add 40 μL of thrombin into each tube to initiate clotting (fibrin formation). The resulting concentrations will be ~150 nm thrombolytic, 2 UN·mL^−1^ thrombin, and 0–40 μm fibrin.
5. Incubate the tubes at 37 °C for 60 min to ensure complete clot formation.
6. Centrifuge the tubes at 4 °C and 20 000 ***g*** for 30 min to sediment clots.
7. Transfer 10 μL of the supernatant, containing the unbound thrombolytic, into each of at least three separate microplate wells to have technical replicates. Transfer the supernatant from each tube into separate wells.
8. Add 40 μL of Boc‐L‐(*p*‐F)FPR‐ANSN‐H‐C_2_H_5_ into each microplate well and immediately proceed with the next steps to start data collection as quickly as possible after initiating the enzymatic reaction. Alternatively, Boc‐L‐(*p*‐F)FPR‐ANSN‐H‐C_2_H_5_ can be added into microplate wells already during centrifugation (*Step 6*) and then directly mixed with the obtained supernatant. The resulting Boc‐L‐(*p*‐F)FPR‐ANSN‐H‐C_2_H_5_ concentration in the microplate well will be 200 μm.
9. Seal the microplate with an adhesive film to prevent evaporation.
10. Insert the sealed microplate into the microplate reader and start data collection with the parameters set in *Step 1* (Fig. 6A).
11. Calculate residual enzymatic rates at each fibrin concentration, proportional to the amount of unbound thrombolytic, by determining the slope of the linear fit of time‐dependent fluorescence curves. Average values obtained for replicates.
12. Transform enzymatic rate values to fibrin‐bound fraction values (*Bind*) using Eq. 5 where *v*_0_ corresponds to the rate measured for the supernatant in the absence of fibrin and *v*_F_ corresponds to the rate measured for the supernatant in the presence of fibrin at the concentration *F*.
13. The dependence of the fibrin‐bound fraction (*Bind*) on the applied fibrin concentration [*fibrin*] (0–40 μm) should provide an increasing hyperbolic trend that can be fit with the corresponding Eq. 6 to determine the desired dissociation constant *K*_d_ (inverse value of fibrin affinity) and limiting fibrin binding capacity *Bind*_lim_ (close to 100%) (Fig. 6B). Semi‐automated data analysis can be performed using File S2.

(5) $$\text{Bind}=\frac{v_{0}-v_{F}}{v_{0}}\cdot 100\%$$

(6) $$\text{Bind}=\frac{{\text{Bind}}_{\lim}\cdot \left[\text{fibrin}\right]}{K_{d}+\left[\text{fibrin}\right]}$$

### Inhibition resistance assay

**Fig. 7:**
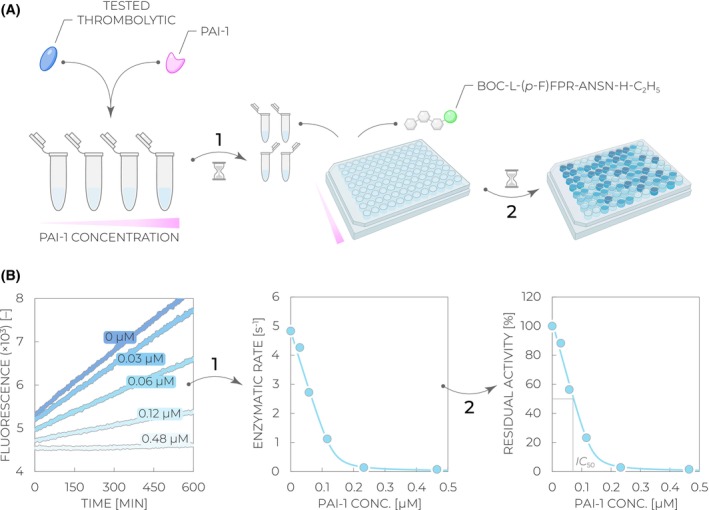
Workflow of the inhibition resistance assay. (A) A tested thrombolytic is mixed with increasing plasminogen activator inhibitor 1 (PAI‐1) concentrations and incubated to reach equilibrium (*Step 1*). Thereafter, the individual samples are mixed with the direct fluorogenic substrate Boc‐L‐(*p*‐F)FPR‐ANSN‐H‐C_2_H_5_. The reaction is monitored via the fluorescence increase with time (*Step 2*). (B) Collected fluorescence kinetic curves at varied PAI‐1 concentrations (dark to light blue) are used to determine residual activities (slopes of the fluorescence increase) and plotted against the used PAI‐1 concentration (*Step 1*). The values are subsequently relativized to the activity of a non‐inhibited thrombolytic, and the desired value of the half‐maximal inhibitory concentration IC_50_ is obtained by fitting the concentration dependence with the equation for a tightly bound inhibitor (Eq. 7).

1. Pre‐incubate the microplate reader to 37 °C and preset all the parameters to have it ready for data collection once the reaction is initiated—excitation 350 nm, emission 470 nm, bottom‐read position, measurement interval 60 s, total measurement time 2–4 h.
2. Dilute the starting material using the assay buffer to obtain 250 μm Boc‐L‐(*p*‐F)FPR‐ANSN‐H‐C_2_H_5_ and 0.75 μm thrombolytic enzyme. Prepare a dilution series of PAI‐1 ranging from 0 to 2.5 μm (at least six concentration points).
3. For each PAI‐1 concentration, add 32 μL into separate Eppendorf tubes and mix each sample with 8 μL of the tested thrombolytic. The resulting concentrations will be 150 nm thrombolytic and 0–2 μm PAI‐1.
4. Incubate the tubes at 37 °C for 60 min while shaking at 180 rpm to reach equilibrium.
5. Transfer 10 μL of the inhibition mixture into each of at least three separate microplate wells to have technical replicates. Transfer the mixture from each tube into separate wells.
6. Add 40 μL of Boc‐L‐(*p*‐F)FPR‐ANSN‐H‐C_2_H_5_ into each microplate well and immediately proceed with the next steps to start data collection as quickly as possible after initiating the enzymatic reaction. Alternatively, Boc‐L‐(*p*‐F)FPR‐ANSN‐H‐C_2_H_5_ can be added into microplate wells during the tube incubation (*Step 4*) and then directly mixed with the obtained inhibition mixtures. The resulting Boc‐L‐(*p*‐F)FPR‐ANSN‐H‐C_2_H_5_ concentration in the microplate well will be 200 μm.
7. Seal the microplate with an adhesive film to prevent evaporation.
8. Insert the sealed microplate into the microplate reader and start data collection with the parameters set in *Step 1* (Fig. 7A).
9. Calculate residual enzymatic rates at each PAI‐1 concentration by determining the slope of the linear fit of time‐dependent fluorescence curves. Average values obtained for replicates.
10. Plot the dependence of the residual enzymatic rate *v*_i_ on the total applied PAI‐1 concentration c_I_ (0–2 μm) and calculate the half‐maximal inhibitory concentration IC_50_ using the tight binding inhibition Eq. 7, where *c*_E_ corresponds to the total thrombolytic enzyme concentration (0.15 μm) and *v*_0_ corresponds to the enzymatic rate in the absence of the inhibitor (Fig. 7B). Semi‐automated data analysis can be performed using File S3.

(7) $$c_{I}=c_{E}\cdot \left(1-\frac{v_{i}}{v_{0}}\right)+\left({\mathrm{IC}}_{50}-\frac{c_{E}}{2}\right)\cdot \left(\frac{v_{0}}{v_{i}}-1\right)$$

## Tips and Tricks

### Fluorogenic substrate handling and storage

1. Stock solutions of D‐VLK‐AMC and AMC are typically reconstituted in DMSO. After the reconstitution, prepare small aliquots and store them at −20 °C. Avoid repeated freeze–thaw cycles and keep them in a dark environment.
2. Keep working stock aliquots at room temperature—not on ice—to prevent their solidification (the melting point of DMSO is 19 °C).
3. We tested up to 3% residual amount of DMSO in the reaction assay, and it did not interfere with the enzymatic activity, so it can be safely used.
4. Spontaneous hydrolysis and/or photobleaching of the substrate may occur. In that case, we recommend including proper negative controls (fluorogenic substrate without the tested thrombolytic and/or plasminogen) and correcting/subtracting the resulting kinetic curves accordingly.

### Fibrinogen source, reconstitution, and storage

1. If delivered lyophilized, reconstitute fibrinogen in water only and at 37 °C while stirring to ensure complete dissolution. Then store at room temperature for a couple of days or as flash‐frozen aliquots for more than a year. Reconstitution in buffers, storage at 4 °C, and repeated freeze–thaw cycles should be avoided to prevent fibrinogen clumping.
2. Make sure you always use “plasminogen‐free” (= “plasminogen‐depleted”) fibrinogen for the *Fibrin binding assay*; otherwise, traces of plasminogen get converted to plasmin and hydrolyze the whole fibrin clot during the incubation step.
3. In contrast, the *Fibrinolysis activity assay* requires ‘common’ fibrinogen; otherwise, no fibrinolysis occurs. Using ‘plasminogen‐free’ fibrinogen can be compensated for by adding a small amount of plasminogen, but such a setup is unnecessarily more tedious and more costly.

### Plasmin(ogen) source and stability

1. Always double‐check the quality and reliability of the sample source and purification method, including batch‐to‐batch variations. Based on our experience, some commercial human‐isolated products contain a substantial portion of interfering contaminants, and various batches may provide very different results. At worst, the protein may not be present in the sample at all.
2. Keep reconstituted/thawed aliquots on ice or at 4 °C at all times to prevent gradual plasminogen activation, significantly biasing the collected data. This is especially critical for the plasminogen‐containing ‘Master Mix’ to prevent D‐VLK‐AMC conversion before the start of the data collection—prepare it shortly before the actual measurement.
3. Due to this gradual plasminogen activation problem, do not store aliquots for longer periods in liquid form. Flash‐frozen aliquots are stable for more than a year. We recommend using freshly reconstituted/thawed aliquots for each measurement campaign.

### Assays modification and components substitution

1. If only absorbance detection is available, the fluorogenic substrates D‐VLK‐AMC and Boc‐L‐(*p*‐F)FPR‐ANSN‐H‐C_2_H_5_ can be substituted with the chromogenic substrates D‐VLK‐pNA (S‐2251) and H‐D‐IPR‐pNA (S‐2288), respectively. The required concentrations may vary and may need further optimization. The activity is monitored by absorbance at 410 nm, but the resulting curves are analyzed in exactly the same way.
2. If no temperature‐controlled reader is available, plates can be kept in a shaking incubator and manually placed to a reader at certain time points (e.g., every 5 min). Although less precise results with fewer time points and more tedious work are obtained, the final values are valid enough. Measurements directly at room temperature are possible but may be less relevant to physiological conditions.
3. For the *Fibrin binding* and *Inhibition resistance assays*, the temperature of kinetic data acquisition typically does not affect the final values as long as all the samples are kept at the same temperature. Therefore, data collection at room temperature is a valid approach, but prolonged kinetic time windows may be required.
4. When analyzing a large number of samples, use ideally a microplate reader with a built‐in dispenser or at least an electronic pipette with the dispensing function for the addition of the last component to minimize the dead time of the measurement. Manual pipetting of the whole 96‐well plate creates a significant time delay between the first and the last well, leading to biased activity values. If manual pipetting is unavoidable, divide samples into smaller batches.
5. Excitation and emission wavelengths during kinetic data collection can be slightly adjusted outside their optima if different filters are available. We have routinely used 360/460 nm excitation/emission filters with no impact on sensitivity, background, or data scatter.
6. Residual thrombolytic activities/amount in the *Fibrin binding* and *Inhibition resistance assays* can be alternatively monitored by substituting Boc‐L‐(*p*‐F)FPR‐ANSN‐H‐C_2_H_5_ with plasminogen and D‐VLK‐AMC (as in the *Enzymatic activity assay*), and the same final results should be obtained. However, such an arrangement makes the assay unnecessarily more expensive and tedious, and also makes the subsequent data analysis more complicated.
7. The *Fibrinolysis activity assay* is a simplified and more reproducible version of the previously published CloFAL assay where whole plasma is used [29]. If desired, the herein presented protocol can be modified accordingly by substituting fibrinogen and thrombin with a plasma sample. Data collection and analysis are then performed still in the same way.

### Experimental setup and aspects of the assays

1. The presence of CaCl_2_ and Tween 80 in the assay buffer is essential for effective coagulation and minimized tube adsorption, respectively, so do not omit them in your assays.
2. ‘No stimulation’ sample in the *Fibrin(ogen) stimulation and selectivity assay* corresponds to the same experimental setup as in the *Enzymatic activity assay*, so both activity and stimulation/selectivity parameters are typically measured in one run.
3. *Fibrinolysis activity assay* provides a global assessment of thrombolysis efficiency as a combination of multiple parameters while the *Enzymatic activity assay* determines specifically the rate of plasminogen activation and stimulation/selectivity factors. Their use depends on the problem to be addressed—while the former one better translates to the overall thrombolysis performance *in vivo* and on *ex vivo* clots, the latter one provides a better understanding of the thrombolytic molecular principles.
4. Although the concentrations of individual components in the *Fibrinolysis activity assay* were optimized to provide a plateau/lag between the clotting and the fibrinolysis phases, significantly more active variants may exhibit lysis initiation already before complete clotting. If this is the case, adjust the assay conditions accordingly—for example, decrease the thrombolytic concentration. Alternatively, use the “ClotLysisCL” online tool within the Shiny App framework, which can determine the value of *t*_50_ from full datasets even with no lag phase [30].
5. Eppendorf tubes can heat up significantly during the centrifugation step (*Step 6*) of the *Fibrin binding assay*. Make sure you use a refrigerated centrifuge set to a low temperature based on the protocol; otherwise, clot integrity and thrombolytic activity may get impaired.
6. ‘Bottom‐read’ positions of the excitation laser and emission detector of the microplate reader are preferred to avoid ‘top‐read’ signal interference and scatter due to condensed drops on the covering microplate film.
7. Thorough shaking during data collection is especially critical for the *Fibrinolysis activity assay* to form a homogeneous fibrin polymer and obtain reproducible results.

## Case study

**Fig. 8:**
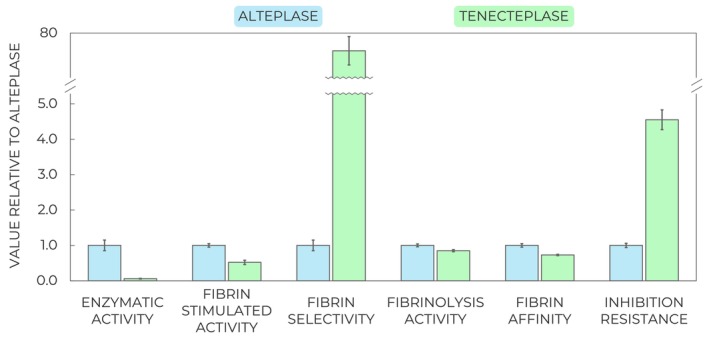
Comparison of key biochemical properties determined for the benchmark thrombolytic enzymes, alteplase and tenecteplase, using the assays featured in this protocol. While tenecteplase yields a low basal enzymatic activity with no stimulation, the presence of fibrin substantially stimulates the activity, resulting in increased fibrin selectivity. Tenecteplase is also more resistant to inhibition, explaining its beneficial properties in animal testing and clinical trials. The error bars correspond to combined standard errors of three technical replicates averaging and data fitting uncertainty.

We applied the aforementioned assays to the most popular benchmark thrombolytic proteins in clinical research, alteplase and tenecteplase, to demonstrate the power of applying the combination of all the provided methods. The results (Fig. 8) showed that the basal enzymatic rate of tenecteplase was very low, seemingly concluding an unappealing thrombolytic protein. However, tenecteplase activity was substantially boosted by fibrin, so its fibrin‐stimulated and fibrinolysis activities nearly reached alteplase, thanks to the significant fibrin selectivity. Together with the identified increased inhibition resistance, this translates to improved half‐life and decreased bleeding risks, explaining the benefits of tenecteplase observed in previous studies [20, 37, 38, 39, 40]. Such an outcome validates the robustness and relevance of the herein presented biochemical assays and clearly manifests their added value for better understanding and selection of the best thrombolytic candidates before any follow‐up *in vivo* trials.

## Conflict of interest

The authors declare no conflict of interest.

## Author contributions

MT and AS optimized the protocols and performed the case study experiments. MT wrote the initial draft of the manuscript and prepared and edited the figures. MT, ZP, and JD conceived and designed the study. ZP and JD supervised the project involving the case study experiments and the corresponding data analysis. All authors contributed to reviewing and editing the manuscript and reviewing the figures.

## Supporting information


**File S1.** Excel datasheet for semi‐automated analysis of enzymatic activity, fibrin(ogen) stimulation, and fibrin selectivity data. The file provides a step‐by‐step guide for easy and user‐friendly data analysis.

**File S2.** Excel datasheet for semi‐automated analysis of fibrin binding data. The file provides a step‐by‐step guide for easy and user‐friendly data analysis.

**File S3.** Excel datasheet for semi‐automated analysis of inhibition resistance data. The file provides a step‐by‐step guide for easy and user‐friendly data analysis.

## Data Availability

The data that support the findings of this study are available from the corresponding author upon reasonable request.
